# Centrosomal Actin Assembly Is Required for Proper Mitotic Spindle Formation and Chromosome Congression

**DOI:** 10.1016/j.isci.2019.04.022

**Published:** 2019-04-28

**Authors:** Matthias Plessner, Julian Knerr, Robert Grosse

**Affiliations:** 1 Institute of Pharmacology, University of Freiburg, Albertstr. 25, 79104 Freiburg, Germany; 2 CIBSS - Centre for Integrative Biological Signalling Studies, University of Freiburg, Schänzlestr. 18, 79104 Freiburg, Germany

**Keywords:** Biological Sciences, Cell Biology, Organizational Aspects of Cell Biology, Functional Aspects of Cell Biology

## Abstract

Cytoskeletal cross talk between actin filaments and microtubules is a common mechanism governing the assembly of cellular structures, i.e., during filopodia formation or cilia organization. However, potential actin-microtubule interactions during mammalian cell divisions are less well understood. At mitotic entry, centrosomes propagate the formation of the mitotic spindle, thereby aligning individual chromosomes to the metaphase plate, a process coined *chromosome congression*. Here, we identify actin filament assembly spatially defined at centrosomes contemporaneously with spindle microtubules forming during prometaphase. We show that pharmacological Arp2/3 complex inhibition as well as overexpression of the Arp2/3 complex inhibitory protein Arpin decreased spindle actin. As a consequence, mitotic spindle formation is impaired, which resulted in disorganized chromosome congression and ultimately mitotic defects in non-transformed cells. Thus centrosomal Arp2/3 complex activity plays a role in the maintenance of genomic integrity during mitosis.

## Highlights

• Spindle actin assembles upon prometaphase at centrosomes
• Spindle actin is branched and precedes kinetochore fibers
• Arp2/3 complex assembles spindle actin
• Inhibition of Arp2/3 complex during mitotic entry impairs mitotic spindle formation

## Graphical Abstract

**Figure:**
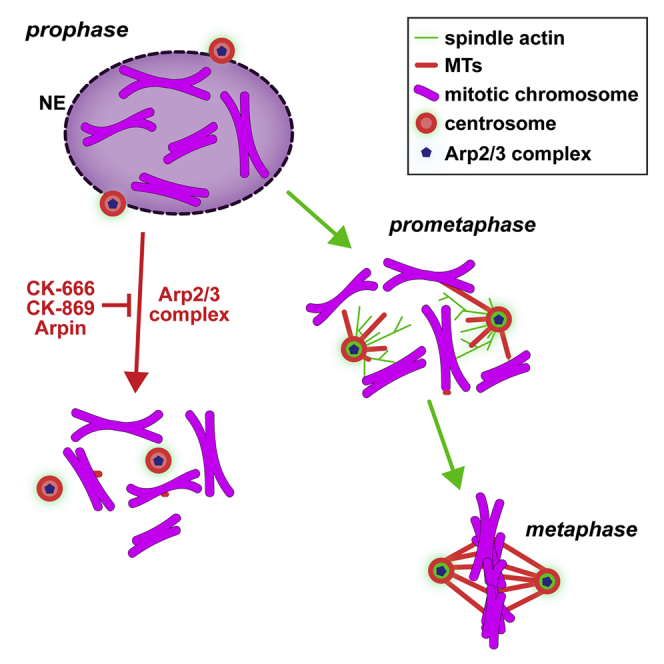

## Introduction

In eukaryotic cells, the cytoskeleton is composed of different components, such as actin, intermediate filaments, microtubules (MTs), or septins, which are assembled into distinct intracellular structures based on local signaling events. Although these structures were thought to be of singular composition, recent progress uncovered evidence for coordinated assembly, especially among actin and MTs (Dogterom and Koenderink, 2018). Such coordinated assembly mechanisms have physiological roles in the control of neuronal cell motility and protrusions (Okada et al., 2010, Henty-Ridilla et al., 2016, Zhao et al., 2017), lymphocyte polarity (Obino et al., 2016), as well as amyotrophic lateral sclerosis (Henty-Ridilla et al., 2017).

Major cytoskeletal remodeling represents a key feature of mitotic entry and is initiated by the disassembly of interphasic structures to facilitate the formation of the mitotic spindle and cytokinetic ring for the segregation of chromosomes and division of the cell body. Mitotic spindles are formed by either centrosomal or chromatin- or MT-based nucleation and polymerization of MTs (Roostalu and Surrey, 2017), but only centrosomal assembly mechanisms produce a symmetric structure of kinetochore fibers necessary for proper allocation of sister chromatids to daughter cells. Interestingly, centrosomes (microtubule-organizing centers, MTOCs) are also able to serve as a template to promote filamentous actin (F-actin) assembly *in vitro* (Farina et al., 2016), whereas actin and MT dynamics are mutually modulated by a variety of factors, i.e., the actin-binding protein profilin (Henty-Ridilla et al., 2017), MT plus-end-binding proteins (i.e., CLIP-170) (Henty-Ridilla et al., 2016), and others such as formins (Ishizaki et al., 2001, Wade, 2007). Herein, a spindle-based accumulation of actin filaments, myosins, and other actin-binding proteins has been reported in several model organisms (Gawadi, 1971, Forer and College, 1979, Maupin and Pollard, 1986, Wu et al., 1998, Yu et al., 2006, Schuh and Ellenberg, 2008, Sandquist et al., 2011, Chan et al., 2014). Furthermore, fission yeast appears to be able to undergo nuclear division in the absence of MTs (Castagnetti et al., 2010), and particularly in *Xenopus laevis* oocytes, actin filaments appear to influence spindle length in anaphase (Woolner et al., 2008). Such filaments have also been discussed to be part of the spindle matrix in germline cells, a structural scaffold enabling chromosome segregation in the later anaphase (Forer et al., 2008, Johansen et al., 2011, Mogessie and Schuh, 2017). However, whether similar actin-dependent processes also occur in somatic cells to aid mitotic spindle formation and thereby drive chromosome congression has not been investigated.

Recent advancements in probing and imaging actin assembly enabled the discovery of several forms of physiological actin filaments in somatic cell nuclei, i.e., as part of the MRTF/SRF transcriptional response (Baarlink et al., 2013), after stimulation of integrins during cell spreading (Plessner et al., 2015), following DNA damage (Belin et al., 2015, Caridi et al., 2018, Schrank et al., 2018), or during re-assembly of the nuclear compartment at mitotic exit (Baarlink et al., 2017, Parisis et al., 2017). Here we describe centrosomal actin filament assembly in somatic cells, which remain discernible from prometaphase until metaphase. For simplicity and concurrent with literature on spindle-associated actin filaments (Wühr et al., 2008, Mogessie and Schuh, 2017), we refer to these filaments as “spindle actin” throughout this article. By co-visualization of MTs and chromosomes, we show that individual actin filaments precede kinetochore fibers and that pharmacological inhibitors and dominant-negative approaches against the Arp2/3 complex decrease spindle actin, impair mitotic spindle formation, and lead to mitotic defects in non-transformed cell lines.

## Results and Discussion

### Formation of Perinuclear and Spindle Actin during Prometaphase

**Figure 1:**
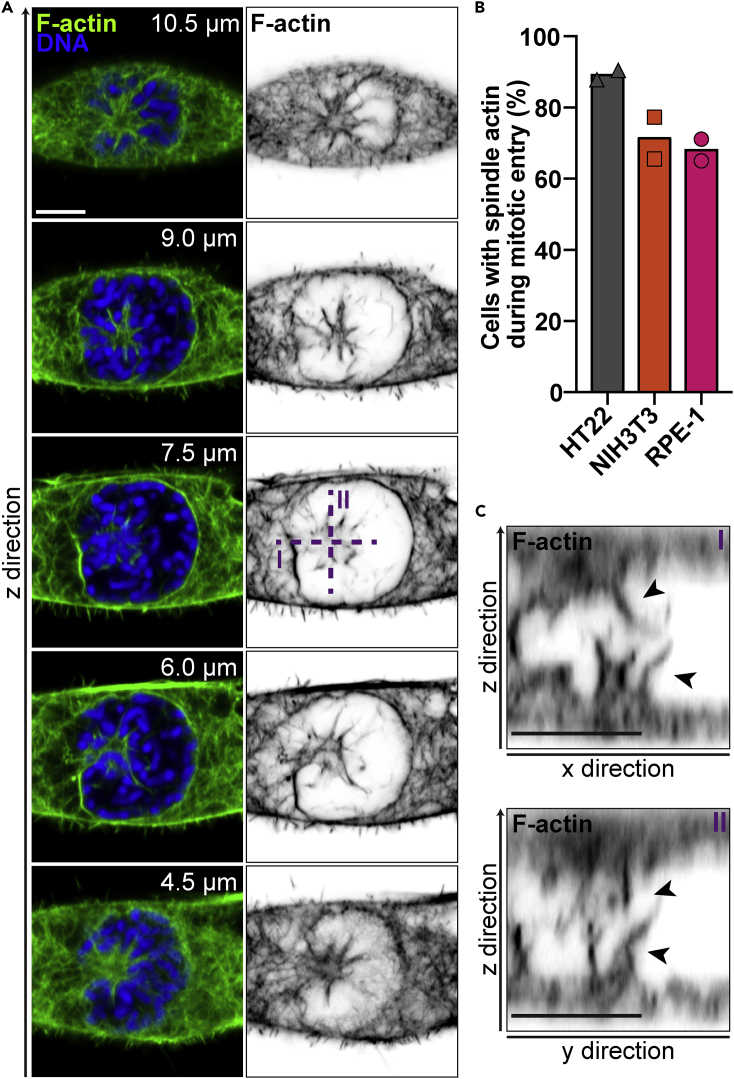
Formation of Perinuclear and Spindle Actin during Prometaphase (A) Confocal scans of glutaraldehyde-fixed NIH 3T3 cells in indicated z direction, stained for F-actin (phalloidin, green, and black and white) and DNA (DAPI, blue). Note the aster-like F-actin structures below and on top of the nucleus (at 6.0 and 9.0 μm) as well as perinuclear F-actin (7.5 μm). Scale bar, 5 μm. (B) Incidence of spindle actin during prometaphase in different mammalian cell lines. Data are shown as mean of two independent experiments together with individual data points; n > 13 cells in prometaphase per data point. (C) Cropped, orthogonal projections of phalloidin staining (black and white) as indicated in (A) (7.5 μm) by dashed, violet lines. Scale bar, 5 μm. Arrowheads indicate F-actin structures protruding into the nuclear compartment.

During our investigation of nuclear actin filaments at mitotic exit in mammalian cells (Baarlink et al., 2017), we noticed prominent F-actin structures during the initial steps of mitotic cell division, which we decided to assess in more detail using glutaraldehyde fixation and phalloidin staining. Most cells showed F-actin at the nuclear envelope as well as two aster-like arrangements of actin filaments during mitotic entry, specifically in prometaphase (Figures 1A and 1B; Video S1). Orthogonal cross sections computed from confocal z stack data (Figure 1C) indicated that these actin structures protrude into the disassembling nuclear compartment.

Video S1. Confocal z stack of NIH 3T3 Cells at Prometaphase with a Step Size of 250 nm, Related to Figures 1A and 1C F-actin, and DNA were labeled by phalloidin and DAPI. Scale bar, 5 μm.

### Spindle Actin Is Branched and Precedes Kinetochore MTs

To study the assembly and dynamics of spindle actin, we imaged mouse fibroblasts (NIH 3T3) and human epithelial cells (RPE-1) upon mitotic entry until formation of a metaphase plate was completed. We labeled endogenous actin using a previously characterized, shuttling (s) anti-actin nanobody (Plessner et al., 2015, Baarlink et al., 2017, Melak et al., 2017), which provides visualization of cytoplasmic and nuclear actin structures. For live-cell MT labeling, we used either SiR-tubulin (Lukinavičius et al., 2014) or stable, low-level mCherry-β-tubulin expression, which was defined by fluorescence-activated cell sorting as the first quartile of bulk fluorescence intensities.

**Figure 2:**
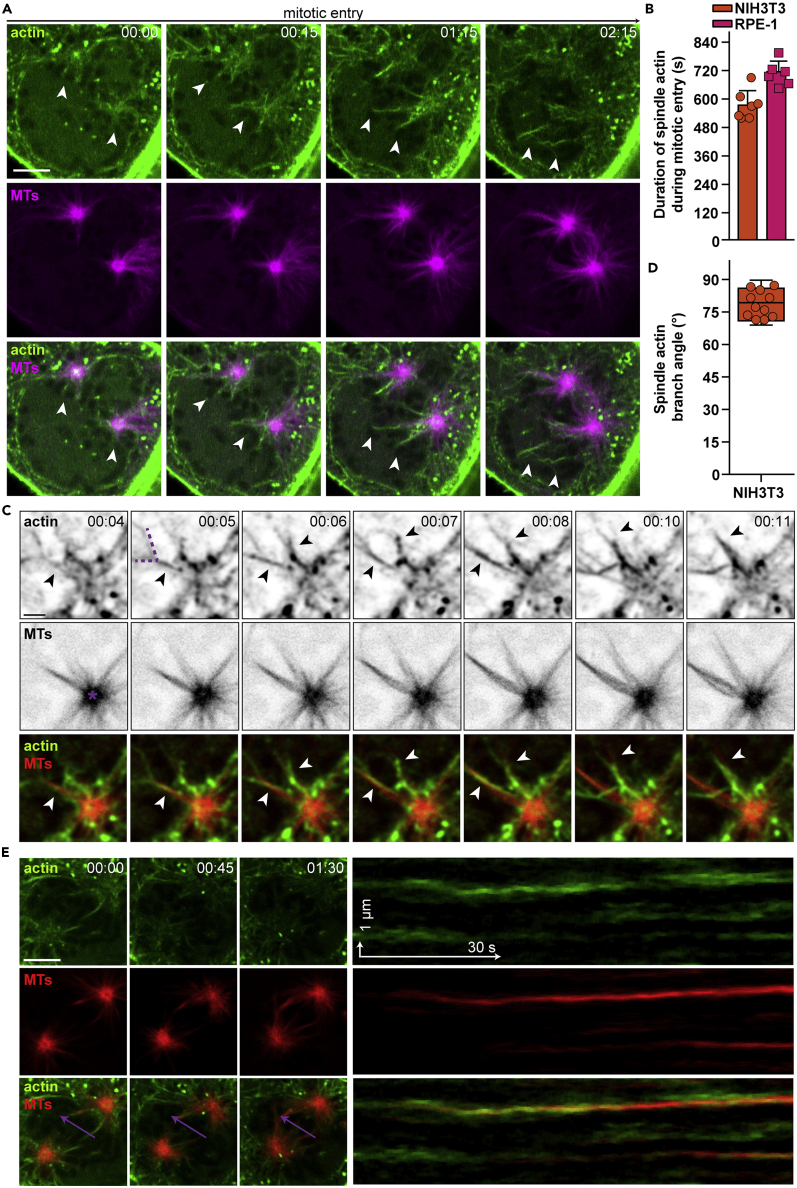
Spindle Actin Is Branched and Precedes Kinetochore MTs (A) Confocal live-cell imaging of RPE-1 cells, stably expressing shuttling actin-chromobody-TagGFP2 (actin, green), stained with SiR-tubulin (MTs, magenta). Spindle actin is assembled at centrosomes and microtubules during formation of the mitotic spindle (arrowheads). Scale bar, 5 μm; time stamp, min:s. (B) Quantification of spindle actin duration in NIH 3T3 and RPE-1 cells. Data are shown as mean + SD of two independent experiments together with individual data points; n > 5 events per cell line. (C) Confocal live-cell imaging of an individual centrosome (asterisk, 00:00) in NIH 3T3 cells during prometaphase, stably expressing shuttling actin-chromobody (actin, green) and mCherry-β-tubulin (MTs, red). Individual actin filaments aligning with forming spindle MTs are indicated by arrowheads. Scale bar, 1.5 μm; time stamp, min:s. (D) Quantification of spindle actin branch angle in NIH 3T3 cells, as indicated in (C) (00:01) by dashed, violet lines. Data are shown as a scatter box plot; n = 11 actin filament branches. (E) Confocal live-cell imaging of NIH 3T3 cells stably expressing shuttling actin-chromobody (actin, green) and mCherry-β-tubulin (MTs, red) during prometaphase. Kymographs corresponding to the violet arrows are shown on the right and illustrate actin assembly before MT polymerization on individual tracks at the equatorial plane. Scale bar, 5 μm; time stamp, min:s.

Live-cell imaging data showed dynamically reorganized actin filaments at centrosomes and along MTs, which were discernible until metaphase (Figure 2A; Videos S2 and S3) for an average duration of about 11 min (Figure 2B). Using improved spatiotemporal resolution, we could visualize branching of actin filaments at a ∼80° angle (Figures 2C and 2D; Video S4) and rapid dynamics within milliseconds. Kymograph analyses of such data exemplified that individual actin filaments precede kinetochore fibers during formation of a metaphase plate (Figure 2E; Video S5), suggesting that these F-actin structures guide assembling MTs.

Video S2. Live-Cell Imaging of NIH 3T3 Cells during Mitotic Entry Labeled for Endogenous Actin (sAC, Green) and MTs (mCherry-β-tubulin, Red), Related to Figure 2A Scale bar, 5 μm; time stamp, min:s.

Video S3. Live-Cell Imaging of RPE-1 Cells during Mitotic Entry Labeled for Endogenous Actin (sAC, Green) and MTs (SiR-Tubulin, Red), Related to Figure 2A Scale bar, 5 μm; time stamp, min:s.

Video S4. Live-Cell Imaging of a Single Centrosome in NIH 3T3 Cells during Mitotic Entry Labeled for Endogenous Actin (sAC, Green), MTs (mCherry-β-tubulin, Red), and DNA (SiR-DNA, Magenta), Related to Figure 2C Scale bar, 1.5 μm.

Video S5. Live-Cell Imaging of NIH 3T3 Cells during Mitotic Entry Labeled for Endogenous Actin (sAC, green) and MTs (mCherry-β-tubulin, Red), Related to Figure 2E Scale bar, 5 μm; time stamp, min:s.

### The Arp2/3 Complex Nucleates Spindle Actin, and Spindle Actin Is Required for Mitotic Spindle Formation

**Figure 3:**
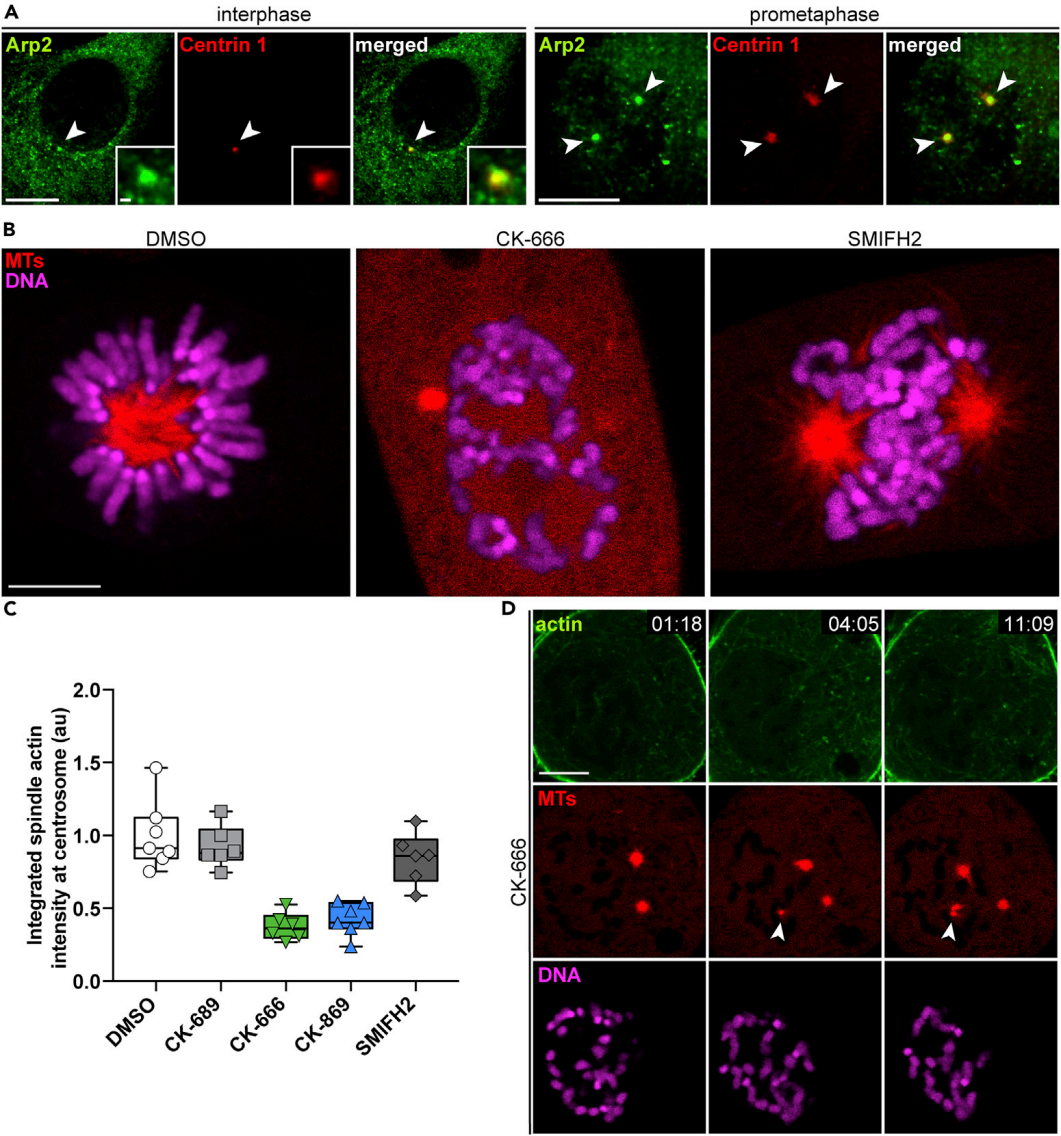
The Arp2/3 Complex Nucleates Spindle Actin, and Spindle Actin Is Required for Mitotic Spindle Formation (A) Centrosomal Arp2/3 complex localization in inter- and prometaphase. NIH 3T3 cells were stained with anti-Arp2 (green) and Centrin 1 (red). Arrowheads indicate centrosomes, and the individual centrosome in interphase is shown as a magnification. For prometaphase, a maximum intensity projection was calculated to show both centrosomes in a single image. Scale bar, 10 μm or 1 μm (magnification). (B) Chromosome congression in NIH 3T3 cells stably expressing mCherry-β-tubulin (MTs, red). Cells were stained with SiR-DNA (magenta) and treated with indicated inhibitors. See also Videos S6, S7, and S8. Scale bar, 5 μm; time stamp, min:s. (C) Quantification of integrated sAC-TagGFP2 fluorescence intensities at centrosomes with indicated treatments during prometaphase. Data are normalized to DMSO and presented as box plots; n = 6 (CK-689, CK-666, SMIFH2) or 7 (DMSO, CK-869) events from two independent experiments. (D) Live-cell imaging of NIH 3T3 cells labeled as in (B) treated with CK-666 during mitotic progression. Note the lack of centrosome-mediated MT assembly, whereas chromatin-mediated MT nucleation is not affected (white arrowheads). Scale bar, 5 μm; time stamp, min:s.

Next, we were interested in identifying the nucleation factors responsible for driving spindle actin assembly. Owing to the apparent branching of actin filaments (Figure 2E), we presumed a role for the Arp2/3 complex. Indeed, centrosomal localization of the Arp2/3 complex subunit Arp2 during prometaphase could be readily detected by immunostaining (Figure 3A). To assess Arp2/3 complex loss of function, we applied the small-molecule Arp2/3 complex inhibitors CK-666 and CK-869 (Hetrick et al., 2013) to cells at mitotic entry while visualizing changes in actin and MT dynamics in real-time by confocal microscopy. Pharmacological inhibition of the Arp2/3 complex, but not of formin actin nucleators (SMIFH2) (Rizvi et al., 2009), resulted in robustly reduced spindle actin levels at centrosomes compared with control cells (DMSO or CK-689) (Figure 3C; Videos S6, S7, and S8). Surprisingly, co-labeling of MTs revealed extensive defects in centrosomal spindle formation after Arp2/3 complex inhibition (Figure 3B), whereas chromatin-mediated spindle formation still proceeded (Figure 3D and Video S9). We also observed striking defects in chromosome congression (Figure 3B), indicating a role for the Arp2/3 complex in maintaining genomic integrity throughout mitosis.

Video S6. Live-Cell Imaging of NIH 3T3 Cells during Mitotic Entry Labeled for Endogenous Actin (sAC, Green), MTs (mCherry-β-tubulin, Red), and DNA (SiR-DNA, Magenta) Treated with DMSO at 00:00, Related to Figure 3B Scale bar, 5 μm; time stamp, min:s.

Video S7. Live-Cell Imaging of NIH 3T3 Cells during Mitotic Entry Labeled for Endogenous Actin (sAC, Green), MTs (mCherry-β-tubulin, Red), and DNA (SiR-DNA, Magenta) Treated with CK-666 at 00:00, Related to Figure 3B Scale bar, 5 μm; time stamp, min:s.

Video S8. Live-Cell Imaging of NIH 3T3 Cells during Mitotic Entry Labeled for Endogenous Actin (sAC, Green), MTs (mCherry-β-tubulin, Red), and DNA (SiR-DNA, Magenta) Treated with SMIFH2 at 00:00, Related to Figure 3B Scale bar, 5 μm; time stamp, min:s.

Video S9. Live-Cell Imaging of NIH 3T3 Cells during Mitotic Entry Labeled for MTs (mCherry-β-tubulin, Red), and DNA (SiR-DNA, Magenta) Treated with CK-666, Related to Figure 3D Note the chromatin-mediated assembly of MTs. Scale bar, 5 μm; time stamp, min:s.

### Spindle Actin Inhibition Results in Mitotic Defects

**Figure 4:**
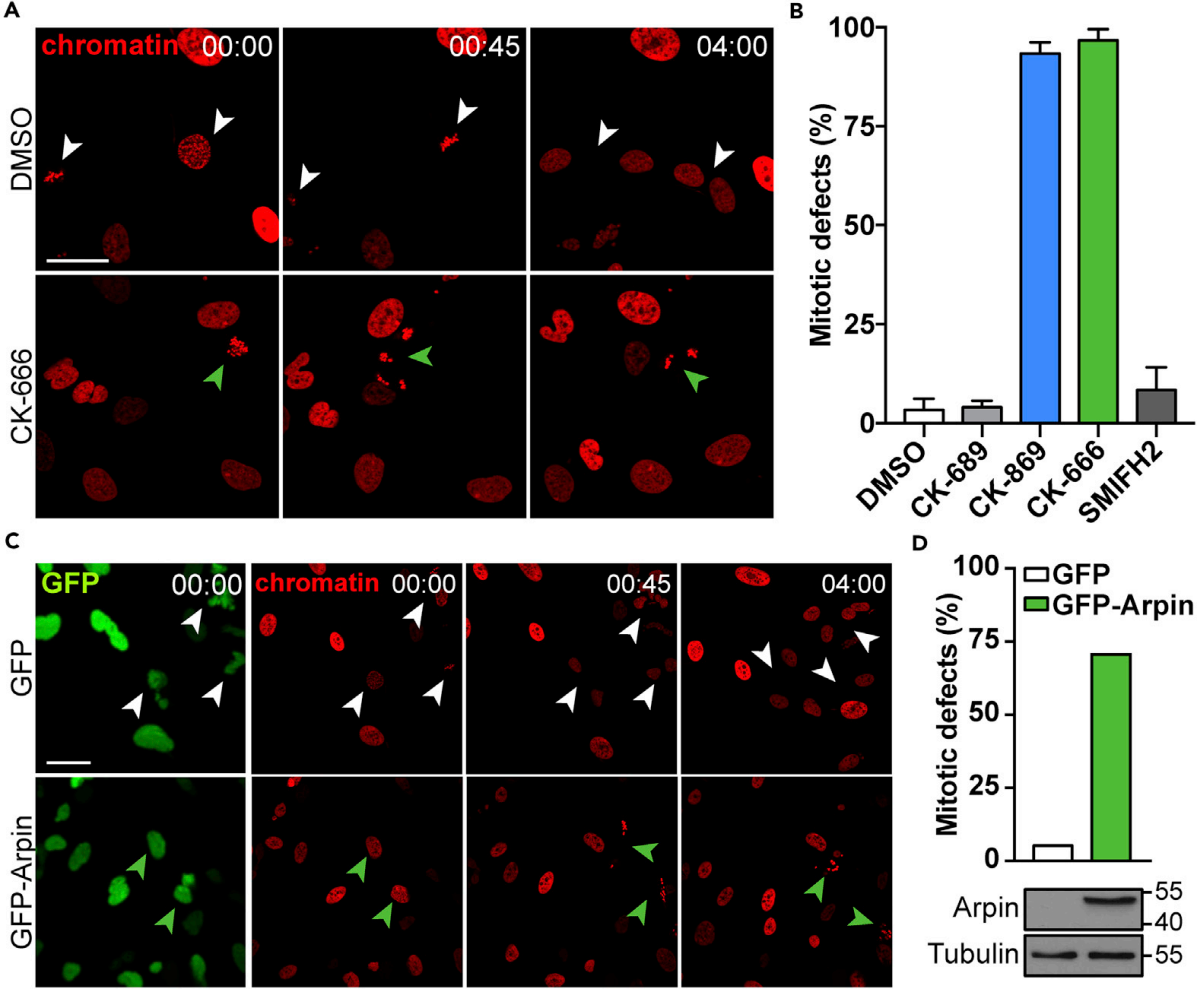
Spindle Actin Inhibition Results in Mitotic Defects (A) Image examples of synchronized RPE-1 cells, stably expressing the chromatin marker H2B-mCherry (red) and treated with indicated compounds at 00:00. Mitotic events are indicated by arrowheads. Scale bar, 25 μm; time stamp, h:min. (B) Quantification of mitotic defects as shown in (A). Data are presented as mean + SD; n > 15 mitotic events, pooled from three independent experiments. (C) RPE-1 cells, stably expressing the chromatin marker H2B-mCherry (red) were transfected with GFP or GFP-Arpin and synchronized at the G2/M border. Image examples show mitotic progression after release of the mitotic block. Mitotic events are indicated by arrowheads. Scale bar, 25 μm; time stamp, h:min. (D) Quantification of mitotic defects as shown in (C). Data are presented as mean + SD; n > 7 mitotic events. Immunoblot indicates expression and molecular weight of Arpin.

To better define the role of the Arp2/3 complex for mitotic progression, we synchronized RPE-1 cells stably expressing the chromatin marker H2B-mCherry with the Cdk1 inhibitor RO-3306 at the G2/M border (Petrone et al., 2016) and applied pharmacological actin nucleation inhibitors after washout of RO-3306. Notably, treatment with CK-666 or CK-869 inhibited chromosome congression and the formation of a metaphase plate (Figure 4A), resulting in severe mitotic defects as observed by scattered chromosomes and micronuclei formation (Figure 4B). To verify the crucial role of the Arp2/3 complex, we utilized the recently identified Arp2/3 complex inhibitory protein Arpin (Sokolova et al., 2017). Interestingly, Arpin overexpression during mitotic entry culminates in a similar phenotype as CK-666 or CK-869 treatment (Figures 4C and 4D). Together, these data point toward a critical role of the Arp2/3 complex for proper mitotic progression.

In summary, our findings reveal a close functional interaction of spindle actin and MTs during prometaphase in dividing, somatic cells. Branched actin filaments are visible at centrosomes, and individual actin filaments precede forming kinetochore fibers. Inhibition of Arp2/3 complex-mediated actin nucleation decreased spindle actin, which subsequently prevented centrosomal MT assembly, whereas chromatin-mediated assembly mechanisms were unaffected. Our data are consistent with previous studies, which implicated centrosomes or MTOCs as actin-organizing centers (Farina et al., 2016). In addition, these observations align with mathematical models explaining the assembly of kinetochore fibers (Forer et al., 2008) and previously reported defects in proliferation after Arp2/3 complex inhibition (LeClaire et al., 2015).

Mechanistically, factors cross-linking actin and MTs, such as CLASP 1 and 2, were already implicated in this process (Konecna et al., 2006) and are thus likely involved in coordinating cytoskeletal filaments for proper mitotic spindle formation. Based on the series of events, in which spindle actin precedes MTs, actin filaments appear to guide individual kinetochore fibers in a spatiotemporal manner due to their faster polymerization rate (Gierke et al., 2012). In addition, kinetochore fiber bundling during chromosome segregation in meiosis is regulated by spindle actin (Mogessie and Schuh, 2017). A potential upstream activator of the Arp2/3 complex is WASH owing to a Cdk1-dependent, activating phosphorylation (Petrone et al., 2016). In fact, this is supported by the notion that we observed Arp2/3 complex also at the centrosome during interphase (Figure 3A), suggesting that a cell-cycle-dependent activation mechanism must occur.

Furthermore, we show a role for Arp2/3 complex activity to promote chromosome congression, which is essential for the correct, subsequent allocation of sister chromatids to daughter cells. As defects in chromosome congression contribute to genomic instability by promoting polyploidy, micronuclei formation, or chromothripsis (Zhang et al., 2015), the impact of deregulated Arp2/3 complex activity during these carcinogenic processes remains an exciting field of future investigations.

### Limitations of the Study

In this study, we describe previously unreported, centrosomal actin structures (spindle actin) during mitotic entry, which are dependent on Arp2/3 complex activity. These actin structures appear to have a function in mitotic spindle formation and subsequent chromosome congression. However, the underlying mechanisms regarding Arp2/3 complex activation at this specific cell cycle stage remain to be elucidated, as well as the involvement of cross-linking proteins or other factors.

## Methods

All methods can be found in the accompanying Transparent Methods supplemental file.

## Supplemental Information

Document S1. Transparent Methods
