# Supplementary material for: Centrosomal Actin Assembly Is Required for Proper Mitotic Spindle Formation and Chromosome Congression
Source: iScience. 2019 Apr 28;15:274–81. doi: 10.1016/j.isci.2019.04.022 (PMC6520610; doi:10.1016/j.isci.2019.04.022)
Supplement: Document S1. Transparent Methods [file mmc1.pdf]

ISCI, Volume *15*

Supplemental Information

Centrosomal Actin Assembly Is Required for Proper Mitotic Spindle Formation and Chromosome Congression

Matthias Plessner, Julian Knerr, and Robert Grosse

Transparent Methods

Cell culture

HT22, NIH3T3 and RPE-1 cells as well as derivatives were maintained in DMEM high glucose supplemented with 10% fetal calf serum (FCS) and 1% Pen/Strep in a 5% CO_2 atmosphere at 37 °C. Stably expressing cell lines were generated using lentiviral transduction (Hinojosa *et al.*, 2017).

Plasmids, antibodies, reagents

H2B-mCherry and sAC-TagGFP2 have been described elsewhere (Baarlink *et al.*, 2017). To generate mCherry-β-tubulin and GFP-Arpin, respective sequences were amplified from human cDNA by PCR and inserted into pmCherry-C1 or pEGFP-C1 (Clontech) using standard molecular cloning techniques. mCherry-β-tubulin was sub-cloned into pWPXL-GFP for generation of lentiviral particles.

anti-GFP (CST, D5.1) and anti-alpha-Tubulin (CST, 11H10) were used for Immunoblotting in Fig. 4d. Immunostaining in Fig. 3a was performed with anti-Arp2 (ab49674, abcam) and anti-Centrin 1 (ab11257, abcam).

SiR-Tubulin and SiR-DNA (Spirochrome) were used at a concentration of 200 nM for 30-45 min to label MTs and DNA, respectively. DMSO (0.1%, Roth), CK-666, CK-689 (100 µM, Sigma), CK-869 (50 µM, Sigma) and SMIFH2 (50 µM, Sigma) were used for drug treatments in Fig. 3 and 4a, b.

Live cell imaging

Except Fig. 3a, all images were generated using a confocal laser scanning microscope (LSM800, Zeiss) with a 63X 1.4 NA objective (except Fig. 4a, c; 20X objective). Cells were cultured in a 5% CO_2 atmosphere at 37 °C using µ-slides (ibidi). Images in Fig. 3a were acquired using a Spinning Disk microscope (Yokogawa, CSU-X1) and a 100X 1.4 NA objective.

Image processing and analysis

Raw data were processed using VisiView (Visitron), ZEN blue (Zeiss), or FIJI (NIH) (Schindelin *et al.*, 2012). Orthogonal cross-sections in Fig. 1c and kymographs in Fig. 2e were computed using FIJI. Branch angle of actin filaments (Fig. 2d) was measured using FIJI. To measure integrated sAC-TagGFP2 fluorescence intensities (Fig. 2b), location of centrosomes was defined by mCherry-β-tubulin fluorescence intensities

(calculated as center of mass), around which a circular ROI (diameter of 2 µm) was placed. sAC-TagGFP2 fluorescence intensities have been subsequently integrated within the circular ROI. Mitotic defects (quantified in Fig. 4b, d) are defined by scattered chromosomes, micronuclei formation or multinucleation.

Synchronization of RPE-1 cells

RPE-1 cells in Figure 4 were synchronized at the G2/M border by application of RO-3306 (10 µM, Sigma) for 16-20 h. Mitotic block was released by washout of RO-3306.

Glutaraldehyde Fixation and Phalloidin Staining

Cells grown on glass coverslips were fixed with 2% glutaraldehyde (Sigma) in PBS for 10 min. F-actin and DNA were stained with phalloidin (100 nM, AF488, Invitrogen) and DAPI in 0.3% Triton X-100/PBS for 48 h at 4 °C.

Immunostaining

Immunostaining was described more detailed elsewhere (Grikscheit *et al.*, 2015). In brief, cells were fixed with 4% PFA in PBS for 10 min. Blocking was performed with 5% goat serum and 1% BSA at RT for 1 h. Cells were then incubated with both primary antibodies at 4 °C for ~24 h. Appropriate secondary antibodies were applied at RT for 2 h.

Statistics

Data were plotted using Prism 7 (GraphPad) as indicated in the respective figure legends.

References

Grikscheit, K., Frank, T., Wang, Y. and Grosse, R. (2015) 'Junctional actin assembly is mediated by Formin-like 2 downstream of Rac1', *Journal of Cell Biology*, 209(3), pp. 367–376. doi: 10.1083/jcb.201412015.

Hinojosa, L. S., Holst, M., Baarlink, C. and Grosse, R. (2017) 'MRTF transcription and Ezrin-dependent plasma membrane blebbing are required for entotic invasion', *Journal of Cell Biology*. Rockefeller University Press, 216(10), pp. 3087–3095. doi: 10.1083/jcb.201702010.

Schindelin, J., Arganda-Carreras, I., Frise, E., Kaynig, V., Longair, M., Pietzsch, T.,

Preibisch, S., Rueden, C., Saalfeld, S., Schmid, B., Tinevez, J. Y., White, D. J., Hartenstein, V., Eliceiri, K., Tomancak, P. and Cardona, A. (2012) ‘Fiji: An open-source platform for biological-image analysis’, *Nature Methods*, 9(7), pp. 676–682. doi: 10.1038/nmeth.2019.
